# Is cholesterol both the lock and key to abnormal transmembrane signals in Autism Spectrum Disorder?

**DOI:** 10.1186/s12944-024-02075-3

**Published:** 2024-04-20

**Authors:** Clifford Lingwood

**Affiliations:** 1 Division of Molecular Medicine, Research Institute, Peter Gilgan Centre for Research and Learning, Hospital for Sick Children, Toronto, ON M5G 0A4 Canada; 2 Departments of Biochemistry and Laboratory Medicine & Pathobiology, University of Toronto, Ontario, M5S 1A8 Canada

**Keywords:** ERAD cholesterol control, Raft lock and key signaling model

## Abstract

Disturbances in cholesterol homeostasis have been associated with ASD. Lipid rafts are central in many transmembrane signaling pathways (including mTOR) and changes in raft cholesterol content affect their order function. Cholesterol levels are controlled by several mechanisms, including endoplasmic reticulum associated degradation (ERAD) of the rate limiting HMGCoA reductase. A new approach to increase cholesterol via temporary ERAD blockade using a benign bacterial toxin-derived competitor for the ERAD translocon is suggested.

A new lock and key model for cholesterol/lipid raft dependent signaling is proposed in which the rafts provide both the afferent and efferent ‘tumblers’ across the membrane to allow ‘lock and key’ receptor transmembrane signals.

## Introduction

Transmembrane signalling is key to cellular homeostasis but is, in particular, the central feature of neurotransmission. The concept of lipid rafts as more ordered, cholesterol enriched microdomains within plasma membranes, first proposed by Simons [1, 2], has put lipid organization at the forefront of membrane biology. While the heterogeneity and dynamics of cellular rafts remain controversial in part, it is clear that cholesterol, which distinguishes eukaryotic membranes, impinges all aspects of membrane signalling. The brain makes its own cholesterol and has the highest tissue content (-25% of the total), mostly in myelin. Cholesterol rafts play an important role in neurotransmission and brain diseases. This is most clear in autism where the disparate risks, including mTOR signaling, may residue under this umbrella.

### Lipid raft composition

**Fig. 1:**
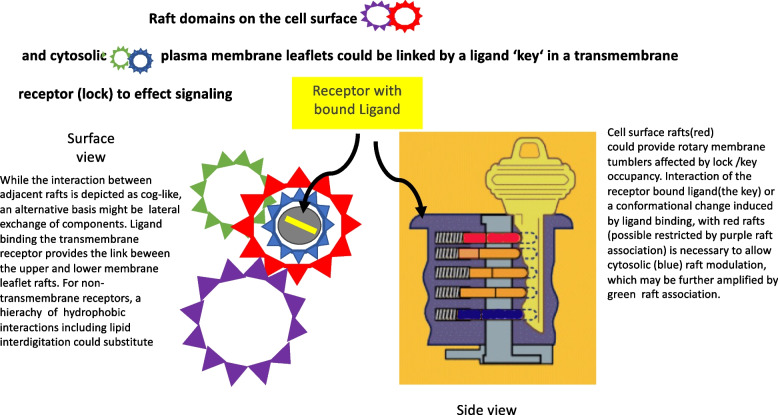
Receptor-ligand: lock-key. Membrane receptor species within lipid rafts may effect transmembrane signaling following ligand binding, via lateral raft-mediated modulation of their membrane environment. Rotation for example, may impact adjacent exoplasmic domains to provide a code for transmembrane domain interactions and subsequent cytosolic signal propagation

Membrane proteins are either associated with or dissociated from, lipid rafts [3]. Membrane proteins are less fluid in raft than non-raft membranes [4]. Lipid membrane anchors [5, 6] hydrophobic matching [7] and protein/protein interactions [8] effect protein partitioning into lipid raft. It is expected that signaling pathways directly involving membrane raft lipids [9, 10] would be more prone to a raft dependent mechanism of transduction (as proposed in Fig. 1). Cholesterol, responsible for their increased order (Lo phase [11], or rigidity) is preferentially found in the outer exoplasmic PM leaflet, but lipid rafts and (lower levels of) cholesterol are also found in the cytosolic membrane leaflet [12–14], although the cytoplasmic species are more unsaturated and less ordered [15]. These opposing leaflet domains may interact in a regulatory manner [16, 17].

The phospholipid species of the exoplasmic and cytoplasmic plasma membrane leaflets are distinct [15]. PC and sphingomyelin are the primary exoplasmic species, while PS and PE are restricted to the cytoplasmic leaflet. Sub-species of PE can interact with cholesterol to form ordered domains [18]. This phospholipid asymmetry, maintained by ABC transporters [19–21], which are in turn, regulated by lipid rafts [22], is one of the first casualties of apoptosis [23].

The planar hydrophobic surface of cholesterol promotes an association with long chain hydrophobic species, particularly GSLs with saturated acyl moieties [24]. The wide array of lipid moieties contained within GSLs regulates their membrane [25] and raft distribution [26, 27]. The interaction of cholesterol can affect the surface conformation of the carbohydrate of GSLs from a membrane perpendicular to membrane parallel format [28]. This can restrict the access to exogenous GSL binding ligands [29]. Depletion of cholesterol can greatly increase the membrane binding of such ligands [30]. In addition, the membrane parallel conformation of GSL carbohydrate can have an ‘umbrella’ effect and mask the hydrophilic(OH) surface of membrane cholesterol [28]. Ligand binding to membrane perpendicular GSL would alter the equilibrium between parallel and perpendicular GSL carbohydrate and could thus promote cholesterol access by reducing the umbrella effect. This in effect, represents a biological transistor. The regulation of membrane receptor clustering after ligand binding, via cholesterol enriched rafts is well studied [31, 32].

### Membrane cholesterol enriched lipid rafts are required for neurological pathways with deficits associated with ASD

Liquid ordered lipid rafts are central to transmembrane signaling in cells [2, 33]. Cholesterol enrichment is the key membrane ordering component in lipid rafts [34, 35]—more rigid heterogeneous domains [36] in the plasma membrane [8] which can recruit, and are required by many transmembrane signaling molecules [37–40]. This includes the mTOR signaling pathway associated with ASD [41, 42], a pathway dependent on both cholesterol [43, 44] and lipid rafts [45], the primary focus of this compendium.

Many studies have implicated low cholesterol (especially ‘good’ cholesterol [46]) as an important factor in ASD [47–52]. Dietary cholesterol supplementation can provide an ASD therapeutic approach [53, 54]. Many diverse genetic factors have been described as risk factors for ASD, [55–62] which has made defining a common mechanism difficult. A disturbance in the cholesterol composition of plasma membrane lipid rafts however, could have a pleiotropic effect on neurological signaling pathways, linking otherwise unrelated signal transduction pathways. Recently it has been proposed that that *Clostridial* metabolites, which primarily inhibit cholesterol biosynthesis, are the primary cause of ASD [63]. Aberrant cholesterol metabolism may predict sensory ASD deficiencies [64]. Protein condensates/phase separation have been implicated as an additional basis for autism [65, 66] and cholesterol can modulate such condensates [67]. Coupling such condensates to lipid ordered membrane domains can define their function [68].

Cholesterol is important in nerve signal transduction [69] and neuronal survival [70]. Cholesterol dependent lipid rafts and receptor protein clustering (densification) [32] are central regulatory components of transmembrane signalling [4], transmembrane signalling is key to nerve synapse function [71] /neurotrophic receptor traffic [72] and synapse function can be defective in ASD [73]. This association has been the subject of excellent review [48, 51, 52].

The mechanism by which low cholesterol could impinge on ASD synaptic and neurotransmission deficiency could be pleiotropic, since many of the ASD associated genes involve cholesterol raft dependent signal transduction/trafficking pathways [49]. Recent studies have further emphasized this linkage. Lipid rafts play key roles in synapse plasticity [74, 75] and have been implicated with an increasing number of signal pathways genetically associated with ASD: mTOR [76, 77], dopamine transport [78, 79], contactin-associated protein-like 2 synapse protein [80], acetyl choline receptor [81] gamma amino butyric acid receptor [82] and glutamate synapses [75, 83–85] and downstream cytosolic reorganization [86]. Nerve membrane receptor clustering is required for synapse function [87]. PCSK9, a regulator of lipoprotein/cholesterol homeostasis and neuronal development/apoptosis [88] has been identified as an ASD risk [89]. Modulation of the lipid raft cholesterol composition is central in Fragile X patients [90] and the rat [91] and mouse model of this ASD [92] and correcting cholesterol levels in part, corrects these differences [92]. Defective neural cholesterol homeostasis is associated with ASD [93].

In addition, cholesterol binding proteins -containing the binding CARC sequence motif [94] found largely in proteins within the exoplasmic membrane leaflet, can mediate interaction with inner membrane proteins containing the mirror CRAC sequence [95], to further amplify the role of cholesterol in transbilayer interactions [96, 97]. Such interactions could similarly be modified by aberrant cholesterol levels.

### Statins and ASD

High cholesterol is associated with coronary problems and oral administration of statins (inhibitors of HMGCoA reductase) is the standard clinical therapeutic stratagem. (These drugs also inhibit protein prenylation since the isoprenoid structures added post-translationally are derived from the same mevalonate pathway [98, 99]). Although the goal, is not to reduce cholesterol below normal and therapeutic efficacy may be limited [100], it is of relevance to consider whether statins have any cholesterol mediated effect on ASD (and other neurological disorders [101]). Moreover, aberrant cholesterol’s association with ASD could include higher levels (as in Rett syndrome [102]) which could also disturb raft dependent signaling. Statin inhibition of cholesterol synthesis can promote axon regeneration [103]. Significantly, statins affect mTOR signalling [43], strongly associated with ASD [42]. Lovastatin treatment of Fragile X rats [104] or Rett syndrome mice [105] prevented cognitive defects. In a double blind randomized, placebo controlled clinical trial in ASD children, simvastatin was found to have a significant beneficial effect monitored by physiological behavioural parameters [106]. In children with neurofibromatosis, a monogenic model for autism, simvastatin also effected brain areas associated with this pathology and showed improved behavioural response in 25% of patients [107]. Drug screening in a drosophila neurofibromatosis model, identified simvastatin as a potential treatment [108], but lovastatin, and not the more apolar simvastatin is effective in the mouse Fragile X model [109]. The mechanistic basis of these results is however, complicated by the dual action of statins on cholesterol and prenylation. Nevertheless prenylation can affect protein-lipid raft partitioning [5, 110] so the effects of reducing cholesterol and prenylation could be related.

### Cholesterol homeostasis

The cellular control of cholesterol biosynthesis is complex, largely defined by a cholesterol sensing mechanism in the endoplasmic reticulum(ER). When cholesterol is low, the ER located transcription factor SREBP(sterol regulatory element binding protein) [111], regulating the transcription of genes required for cholesterol biosynthesis, is transported to the Golgi by SCAP(SREBP cleavage activating protein) [112] for activation by proteolytic cleavage and SREBP then transits to the nucleus to activate the cholesterol biosynthetic genes. Thus, low ER cholesterol stimulates cholesterol levels via gene transcription.

An additional ER cholesterol regulated pathway down regulates cholesterol levels by a post translational mechanism. In the cholesterol biosynthetic pathway, the enzyme, 3-hydroxy-3-methylglutaryl coenzyme A (HMG-CoA) reductase is rate limiting, and one of the additional ways this enzyme levels are regulated is by an unusual process, that of endoplasmic reticulum associated degradation (ERAD). ERAD is the normal cellular quality control mechanism, eliminating nascent misfolded proteins during ER traffic via a chaperone mediated protein unfolding [113], and subsequent transit through the ER translocon (dislocon [114]) to the cytosol for proteosomal degradation, ensuring the dissemination of only correctly 3D folded protein. In a few cases however, the correctly folded protein is also subject to ERAD as a means of control, for example CFTR [115, 116] and HMG-CoA reductase [117]. In terms of HMG-CoA reductase, this pathway is activated via low ER sterol-induced binding to Insig, [118, 119], ubiquitinylation by associated ligases [118–121], to initiate HMGCoA reductase unfolding and ER translocon transit to the cytosolic proteosome for degradation, reducing its cellular expression and thereby, cholesterol biosynthesis [117]. Many ER stress protein mutations are related to ASD [122] which might also impinge such ER regulated cholesterol metabolism.

### Novel ERAD-based means to address hypocholesterolemia

The ERAD pathway is hijacked by many microbial pathogens since it provides a means to access the cell cytosol from the lumen of the ER/Golgi endomembrane system [123]. These include several viruses [124, 125] and cholera and Shiga toxins [126–128]. These toxins enter the endomembrane system by means of their carbohydrate (glycolipid) pentameric B subunit cell surface receptor binding which initiates internalization and retrograde transport to the ER [129]. Here the B subunits separate from the catalytic A subunit. The A subunit contains an N-terminal peptide sequence that mimics an unfolded (misfolded) protein [130], which recruits the ERAD machinery to transmit the A subunit from the ER to the cytosol via the translocon [131], and by avoiding the proteasome, to refold and access its cytosolic target protein (adenylate cyclase for cholera toxin and ribosomal RNA for Shiga toxin). Because of this, cholera toxin has been often used as a tool to probe the mechanisms of ERAD [132–134].

Since the dimensions of the ER translocon accommodate only one protein at a time, we have used this toxin/ERAD hijack as a means to exogenously regulate ERAD [135]. Many genetic diseases are exacerbated by ERAD, in that gene mutations that do not completely inactivate protein function, nevertheless induce minor protein misfolding, and thence ERAD elimination to cause/exacerbate insufficiency disease symptoms [136]. Such genetic diseases include cystic fibrosis, Gauchers Disease, Tay Sachs Disease, Fabry Disease and many more [137]. By mutational inactivation of the toxin A subunit, we generated a benign tool (e.g. mutant cholera toxin- mCT in which the A subunit catalytic activity is removed and does not induce a stress response [138]). This can block (occupy/compete for) the ERAD translocon and thereby allow such partially misfolded but functional nascent mutant proteins (e.g. deltaF508CFTR chloride transporter in cystic fibrosis, N370S glucocerebrosidase in Gaucher) to escape degradation and function to ameliorate deficiency disease symptoms. This system works in cell disease models [135] and mCT is highly effective in a mouse model of CF(delta F508CFTR) to normalize chloride-dependent saliva production [up to > 2 × normal] ([139], a standard index of CFTR function. mCT (rather than other subunit toxins) is the preferred ERAD blockade since the cholera toxin receptor, GM1 ganglioside is expressed on virtually all mammalian cells.

Since normal cholesterol biosynthesis is regulated in part, by ERAD of HMGCoA-reductase, our mCT ERAD blockade approach [135] also offers a potential benign, titratable means to temporarily reduce HMG-CoA reductase degradation to increase cholesterol biosynthesis during hypocholesterolemia. Indeed, blockade of HMGCoA-reductase ERAD has already been shown to increase cholesterol [121]. Furthermore, cholera toxin is able to transit the blood brain barrier [140, 141] and could therefore also modulate neural cholesterol metabolism.

#### Lock and Key receptor binding- only half the mechanism

The concept that protein ligands bind to their membrane receptors by a lock and key molecular complementary mechanism, is well entrenched [142] and validated in molecular biochemistry [143] particularly enzyme mechanisms [144]. However, this is only (less than?) half the living picture. Lock and key essentially only provides insight into the control of ligand/ receptor binding. Such membrane receptors are often recruited to lipid rafts which is essential to their subsequent signal function [145, 146]. The question of how a signal is transmitted is not addressed. The concept of a conformational change is handwaving. Why do downstream transmembrane enzymes etc. become activated, cluster, change? The rest of the lock needs to be considered: the tumblers and escape mechanism.

The eukaryotic membrane is amazingly complex, particularly in its lipid content [15, 147]. Why are so many long chain isoforms made? Cholesterol distinguishes eukaryotes and lipid rafts have revolutionized the way we consider transmembrane signaling. Rafts are more rigid domains in the outer leaflet of the plasma membrane, primarily as a result of their increased cholesterol content [148]. Glycosphingolipids (GSLs) are also key components and the binding of cholera toxin to its glycosphingolipid receptor, GM1 ganglioside, has long been used as a cytochemical marker of lipid rafts [129, 149].

Lipid rafts are heterogeneous [36] but generate a platform which could provide the ‘tumblers’ which determine on or off, cluster or separate, associate or disassociate. What if any, is the rotatory component? Although it took man to invent the wheel, molecular rotation is well described in the mitochondrial and other ATPases as the proton pump mechanism to generate ATP [150] and hence life. However, molecular rotation as a control mechanism in transmembrane signalling has not been considered. When the key opens the lock, are there tumblers? do the tumblers turn? Which way? How far? With whom? In what plane? Is it energy dependent? What are they? A plausible scenario is shown in Fig. 1. Here we propose lipid rafts are the tumblers. The lipid raft ordered domains are considered cogs (delimited by line tension [151]) on either side of the plasma membrane [11] which can rotate around the ligand/receptor complex. In this model, ligand membrane receptor binding engages the aforementioned tumblers for transmembrane coordination of these cogs. In the lock and key schematic (right), the proximal tumbler (red) is the exoplasmic PM raft in which the receptor is embedded, while the distal is a cytosolic leaflet lipid raft(blue). The ligand binding mediated coordination of these *interplanar* rafts induces/amplifies signaling. The species within lipid rafts have restricted translational(lateral) freedom [152, 153] which would aid cohesive rotary lipid raft tumbler signal transmission. The interlocking of exoplasmic and cytoplasmic lipid rafts could be further regulated via additional lateral raft association in the upper or lower bilayer leaflet (Fig. 1). Such lateral raft interactions can serve to recruit the additional downstream components of the signaling pathway. Transmembrane protein receptors could function as the lock to mediate these interplanar raft interactions, while aligning lipid interdigitation [154] could prove a mechanism for peripheral membrane protein raft receptors, GPI anchored proteins [155], or indeed, the interdigitation of raft lipids themselves [10, 154, 156]. Very long chain fatty acid synthesis is considered to facilitate interleaflet lipid interdigitation [157, 158]. The synthesis of such fatty acids has recently been found to be essential in neuronal growth cone lipid rafts and cell polarity [159]. The raft cholesterol content is different between exoplasmic and cytoplasmic membrane lipid rafts [160] and will play a marked role in their “tumbler” function, affected by sterol deficiency (or excess).

To extend the lock and key simile, the cell could be considered as a “safe” with an extremely complex but interrelated membrane “combination”. Cholesterol provides the blueprint for the lipid raft tumblers to enable the various “keys” to access this safe.

## Conclusions

Membrane order is an important player in transmembrane signalling and cholesterol plays a large part in determining membrane order via the dynamic formation of lipid rafts. These rafts can communicate from one membrane side to the other, and this linkage can mediate ligand-membrane receptor binding dependent signal transduction. The defects in cholesterol homeostasis in ASD (and other neurological diseases) suggests this role is particularly important in neural physiology and networks, and increasing cholesterol provides a target for intervention. We suggest a novel mechanism to achieve this increase and propose an addition to the ‘lock and key ‘concept for membrane receptor binding in which cholesterol lipid rafts provide the tumblers to allow and discriminate signals across the membrane.

## Data Availability

No datasets were generated or analysed during the current study.
